# Management of multiple non-magnetic intraocular and intraorbital foreign bodies following a motor vehicle accident: a case report

**DOI:** 10.3389/fmed.2025.1519711

**Published:** 2025-05-23

**Authors:** Shao-lei Han, Ting-ting Wang, Yuan Wen, Lin-bo Liu, Ran Zhao

**Affiliations:** ^1^Department of Ophthalmology, Hebei Eye Hospital, Xingtai, China; ^2^Department of Ophthalmology, Hebei Medical University, Shijiazhuang, China

**Keywords:** intraocular foreign body, intraorbital foreign body, non-magnetic, open-globe injury, prognosis

## Abstract

We report a rare case of multiple non-magnetic intraocular and intraorbital foreign bodies (IOFBs) resulting from a motor vehicle accident. A 29-year-old male presented with sudden vision loss and pain in his right eye after windshield fragments penetrated the eye. Initial examination revealed severe visual impairment. Emergency and subsequent staged surgical interventions were performed to remove multiple glass-like foreign bodies, repair retinal detachment, and ultimately restore visual acuity. Three months after extensive procedures, best-corrected visual acuity significantly improved to 0.2. This case report is crucial as it presents a rare instance of a patient with open-globe injury and multiple non-magnetic IOFBs achieving good visual outcomes despite complex procedures. It underscores the importance of thorough evaluation, phased surgery, and multidisciplinary teamwork in managing such cases, while also indicating the associated difficulties, thereby offering valuable insights for clinical decision-making and future research on severe ocular traumas.

## Introduction

Intraocular foreign bodies, commonly referred to as IOFBs, represent a substantial and critical cause of vision impairment and loss in cases involving open-globe injuries (1–4). These injuries, which occur when the eyeball penetrates or ruptures, have a notable incidence rate, ranging from 18 to 41% (5). The presence of foreign objects within the eye poses significant challenges not only in terms of accurate diagnosis but also in the effective management and treatment of these injuries (6). Frequently, patients with IOFBs require a series of medical interventions and procedures to achieve the best possible visual outcomes, as foreign bodies can cause a range of complications, including inflammation, infection, and further damage to ocular tissues (4, 7–11).

We present a detailed case study of a patient who experienced multiple non-magnetic intraocular and intraorbital foreign bodies as a consequence of a motor vehicle accident. This case underscores the critical importance of conducting a thorough and comprehensive evaluation to identify all foreign objects, as the omission of any one could lead to incomplete treatment and unsatisfactory visual recovery. In managing such complex situations, a phased surgical management strategy is particularly important to ensure gradual removal of all foreign bodies while minimizing further damage to the eye. In this case, we emphasize the importance of multidisciplinary team collaboration and personalized treatment plans in the management of open-globe injuries to improve the patient’s visual prognosis and overall treatment outcomes.

## Case report

**FIGURE 1:**
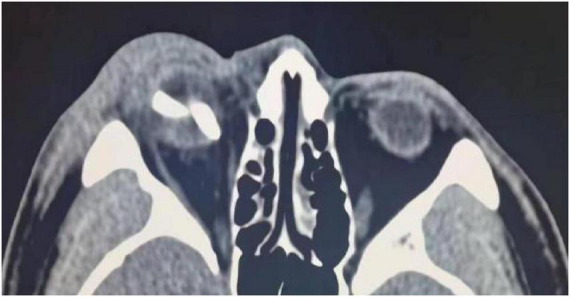
A computed tomography (CT) scan revealed an irregular contour of the right eyeball, indicating the potential presence of intraocular foreign bodies (IOFBs).

A 29-year-old male was referred to our ophthalmology department one day after sustaining a right eye injury in a motor vehicle accident. He reported sudden loss of vision, accompanied by pain and bleeding in the affected eye. The accident involved windshield fragmentation, with some fragments penetrating his right eye. On initial presentation, his visual acuity in the right eye was limited to hand motion at 20 cm. A preliminary examination revealed an irregular contour of the right globe. An urgent computed tomography (CT) scan was performed given the severity of the injury and the potential for retained foreign bodies, and it revealed an irregular contour of the right globe, suggesting the presence of possible intraocular foreign bodies (IOFBs), some of which appeared to be partially extending beyond the eye into the surrounding tissue (Figure 1).

**FIGURE 2:**
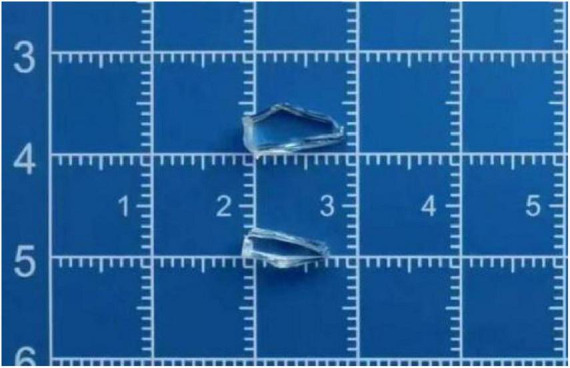
Dimensions of approximately 10 mm in length, 4 mm in width, and 2 mm in thickness, as well as an additional specimen measuring 8 × 3 × 2 mm.

During the preoperative evaluation phase, the patient underwent emergency surgery. The surgery was conducted under general anesthesia and aimed at repairing the right eyeball, extracting intraocular foreign bodies (IOFBs), and reconstructing the eyelid. The surgical team meticulously removed two foreign bodies that resembled glass, with dimensions of approximately 10 mm in length, 4 mm in width, and 2 mm in thickness, as well as another measuring 8 mm × 3 mm × 2 mm (Figure 2).

**FIGURE 3:**
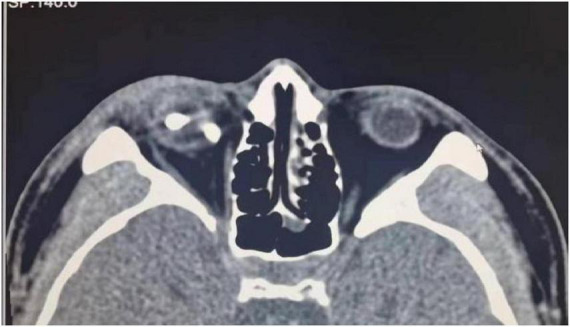
Thirteen days post-surgery, a follow-up computed tomography (CT) scan revealed that some intraocular and intraorbital foreign bodies remained.

**FIGURE 4:**
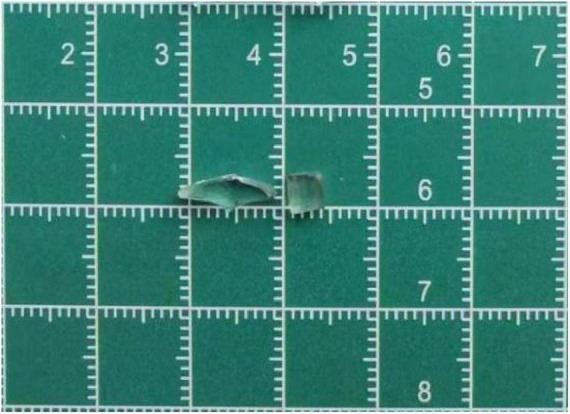
During this surgical procedure, two additional foreign bodies were extracted: an 11 × 3 × 2 mm intraorbital foreign body and a 4 × 3 × 2 mm intraocular foreign body.

In April 2021, subsequent follow-up examinations provided further insight into the patient’s condition. B-scan ultrasonography revealed retinal and choroidal detachment in the right eye. Detachment of the ciliary body in the right eye was identified using ultrasound biomicroscopy (UBM). Functional testing using flash electroretinography (F-ERG) revealed a flat rod response and oscillatory potentials (OPs) in the right eye with significantly diminished amplitudes in other responses. Computed tomography (CT) confirmed the presence of retained foreign bodies in both the eyeball and orbit (Figure 3). Based on these findings, a second surgical intervention was deemed necessary and performed under general anesthesia. This procedure involves pars plana lensectomy and vitrectomy, complex repair of retinal detachment, endolaser photocoagulation, peripheral iridectomy, and removal of intraocular and intraorbital foreign bodies. In addition, silicone oil tamponade was used to support the structure of the eye. During this surgery, two more foreign bodies were extracted: an 11 mm × 3 mm × 2 mm intraorbital foreign body and a 4 mm × 3 mm × 2 mm intraocular foreign body (Figure 4).

By September 2022, the patient underwent removal of the silicone oil. In March 2023, the patient underwent scleral-fixed intraocular lens implantation. Three months after the last surgery, best-corrected visual acuity in the right eye improved to 0.2. The eyelids exhibited no signs of swelling, the conjunctiva was not hyperemic, the cornea remained clear, and the anterior chamber was of normal depth, without any signs of inflammation. The pupil was slightly irregular and lacked light reflex. The intraocular lens was positioned properly. Fundus examination revealed a visible optic disk, absence of foveal reflex, an attached retina, and extensive peripheral laser scars. The intraocular pressure was within the normal range.

This detailed account underscores the complexity and multi-stage nature of the patient’s treatment, highlighting the comprehensive approach taken by the medical team to address sustained severe ocular injuries.

## Discussion

This case report presents a complex scenario involving multiple non-magnetic intraocular and intraorbital foreign bodies resulting from a motor vehicle accident. Such cases pose significant challenges in terms of diagnosis, surgical management, and rehabilitation, particularly owing to the potential for multiple surgeries and prolonged recovery periods.

The presence of non-magnetic foreign bodies, such as glass, complicates the diagnostic process because these materials do not respond to magnetic resonance imaging. CT scans are essential in such cases as they provide critical information on the location, number, and nature of foreign bodies (2, 4, 12). This case highlights the importance of comprehensive imaging for assessing the extent of intraocular and intraorbital involvement, which directly influences surgical planning (13, 14).

In this case, a staged surgical approach was crucial for optimal recovery. Initial surgery focused on emergency globe and eyelid repair and removal of the most readily accessible foreign bodies. The presence of additional retained foreign bodies requires subsequent interventions, including lensectomy, pars plana vitrectomy, and complex retinal detachment repair (15). The successful removal of additional foreign bodies during this stage validates the careful timing and approach, consistent with the findings of He et al. regarding the optimal timing of secondary interventions (16). This approach underscores the necessity of a multidisciplinary team and readiness for multiple procedures to achieve anatomical and functional restoration (9).

Silicone oil tamponade plays a vital role in stabilizing the retinal post-detachment repair. Its use is well-documented in managing complex retinal detachments, especially when associated with significant trauma and multiple foreign bodies (17, 18). This decision aligns with the recent studies by Liu et al. (9), who reported improved outcomes with silicone oil in cases of complex retinal detachment. Although silicone oil can lead to elevated intraocular pressure and other complications, careful monitoring and timely removal, as performed in this case, mitigated these risks (1). The eventual removal of silicone oil without incident indicates prudent timing and effective surgical intervention.

The patient’s visual recovery to an acuity of 0.2 highlights the potential for significant vision improvement, even in severe cases. Scleral-fixated intraocular lens implantation provides optical rehabilitation in the absence of a natural lens. This technique is particularly valuable when standard intraocular lens placement is not possible because of structural damage or absence of the lens capsule.

Despite these improvements, residual visual impairments, such as slight irregularity of the pupil and the absence of light reflexes, indicate the extent of trauma and intraocular damage. This case highlights the need for further advancements in surgical techniques and materials, particularly for handling non-magnetic foreign bodies. Additionally, the integration of postoperative care with rehabilitation services is crucial for optimizing the long-term outcomes.

Future research should explore innovative surgical tools and materials that can further enhance the safety and efficacy of surgeries involving non-magnetic foreign bodies. Additionally, developing protocols that integrate postoperative care with comprehensive visual rehabilitation services is crucial to maximize long-term visual outcomes in patients with similar extensive ocular injuries.

In conclusion, this case exemplifies the complex and layered approach required to manage multiple intraocular and intraorbital foreign bodies. This underscores the importance of thorough evaluation, meticulous surgical planning, and staged interventions to achieve desirable outcomes in patients with extensive ocular trauma. Future studies and technological advancements may provide new insights and improve techniques for managing similar cases, ultimately enhancing the prospects of patient care and recovery.

## Data Availability

The original contributions presented in this study are included in this article/supplementary material, further inquiries can be directed to the corresponding author.
